# Stampede Prevention Design of Primary School Buildings in China: A Sustainable Built Environment Perspective

**DOI:** 10.3390/ijerph15071517

**Published:** 2018-07-18

**Authors:** Kefan Xie, Yu Song, Jia Liu, Benbu Liang, Xiang Liu

**Affiliations:** 1 School of Management, Wuhan University of Technology, Wuhan 430070, China; xkf@whut.edu.cn (K.X.); songyu962@126.com (Y.S.); liangbenbu@whut.edu.cn (B.L.); 2 School of Information and Safety Engineering, Zhongnan University of Economics and Law, Wuhan 430073, China; 3 Institute of Finance, Guangzhou University, Guangzhou 510006, China; 18571530906@163.com

**Keywords:** stampede prevention, sustainable layout, sustainable built environment, dynamic steady ability, step depth

## Abstract

In China, crowd stampede accidents usually take place within crowded areas in middle and primary schools. The crowd stampede risk is particularly related to the architectural design such as the staircase design, the layout of crowded places, obstacles, etc. Through the investigation of building design in several primary schools, the relationship between the sustainable layout of crowded places (e.g., toilets, canteens, playgrounds, staircases) and the crowd stampede risk of students are introduced via agent-based simulations. In particular, different experimental scenarios are conducted on stairs in the primary buildings. The evacuation processes are recorded by video camera and spatial stepping characteristics (e.g., foot clearance, step length, mass center, the distance between the mass center and ankle, and etc.) are extracted from the video. Dynamic steady ability is investigated by adopting the margin of stability, quantified by the instantaneous difference between the edge of the base of support and extrapolated vertical projection of the mass center. Based on the sustainable built environment principles and historical data of students, this paper focuses on an in-depth analysis of the staircase design aiming at preventing the crowd stampede risk.

## 1. Introduction

In China, crowd stampede accidents (CSAs) have occurred within crowded areas in middle and primary schools in recent years [1,2]. With regard to the evacuation in an emergency, the primary school students tend to exhibit an immature mind and low decision-making ability under diverse architectural scenes. They are considered more likely to be involved in dangerous situations. For instance, a 5.7-magnitude earthquake hit Jiangxi, China, on 26 November 2005, and 72 students were injured in a CSA; one student died and 22 students were injured by a CSA at the Experimental Primary School in Puyang, Henan, on 22 March 2017, when students went to the toilet before the examination [3]. According to statistics, CSAs often occur in primary schools located in less developed areas [2].

The CSA shows representative features compared with ordinary accidents. Many causes that may contribute to a crowd stampede have been discussed by previous studies such as the dynamic pedestrian flow in the mass gathering area [4], crowd panic [5], individual collisions [6], obstacles [7], etc. However, the location and triggers of the CSA are usually uncertain and the occurrence is too fast to catch. In addition to on site video surveillance technology and the use of some human trackers [8] to capture the crowd dynamics, stampede prevention building design is addressed as another important pre-control strategy to avoid crowd risks. Studies by Zhao et al. [9] and Li et al. [10] report that with the increase of door width, the total evacuation time decreases nonlinearly within a certain range. The findings presented by Shi et al. [11], Guo et al. [12] and Liao et al. [13] suggest that the internal layout (e.g., multi-exit design, building layout, obstacle location, etc.) guides the evacuation route and affects the total evacuation strategy. Most CSAs usually occur near staircases which are regarded as a particularly special risk site. Pedestrians may pack closer together and even lead to some dangerous effects. The movement and evacuation dynamic of pedestrians on the staircase have been modeled to describe the influence of architectural geometry on the individual motion [2,14,15].

Sustainable design, as a product in this time of a rapid development with the increasing demand for scarce resources, has become a dominant issue in our era [16]. According to the OECD project, the sustainable built environment can be defined as “the building that continues functioning into the future and holds the minimum negative effects without being forced into decline by the exhaustion of key resources on which the architectural system depends [17].” Sustainable building design is related to the natural environment, building layout, and comprehensive settings, which strive for integral aspects including economic (e.g., resource efficiency), social (e.g., harmonization with culture), and environmental performance (e.g., pollution prevention, energy efficiency) [18,19,20]. However, besides in accordance with environmental policies through energy conservation, a sustainable building renovation process considers optimal conditions in security, habit, and comfort through building’s adaptation to the new requirements [21,22]. There remains a need to promote a novel aspect of refurbishment that improves building environments in terms of safety and demands about the special residents, especially those of vulnerable groups (e.g., young and elderly people) [23]. Several studies, such as Koh et al. [24] develops a process of sustainable renovation considering the elderly’s mobility in existing neighborhoods in Singapore or the study developed by Bratucu et al. [25], which combines the safety with the sustainable development in the road construction. Nevertheless, some architectural and human factors (e.g., evolutionary individual characteristics data, dynamical behavior preferences, multiple risks, etc.) are usually overlooked because of energy savings and not comply with the sustainable safety codes, which may affect the long-term practicality of buildings [26,27]. Thus, as a branch of the sustainable built environment, this is a relevant challenge for the stampede prevention sector. It’s necessary to take individual’s habit, custom, and dynamic evolution data into account to continuously reduce the risk of CSAs from a perspective of sustainable safety.

Previous studies on the crowd stampede risk involved in the mass gathering or evacuation within different buildings can be classified into three types. Observation studies typically focus on the non-emergency situation to investigate the daily behavior and walking habit of individuals, which provide a large amount of behavior data to building design [18,28,29]. Experimental studies or evacuation drills select a number of pedestrians to evacuate from a given building in the case of a hypothetical disaster [30,31]. The motion parameters of the pedestrians are recorded by a video camera for later analysis [31]. With the building structure being set in advance, the effect of architectural parameters on the evacuation efficiency of students can be measured accordingly [1]. Simulation approaches are widely adopted to mathematically describe the crowd dynamics and evacuation process. Pedestrians are simulated with the classical theories or models such as the grid-based discrete models [7,32], force-based models [5,10], etc. The basic crowd flow in the building has been modeled utilizing the mentioned methods, such as the bidirectional pedestrian flow in a corridor [33], walking around a bottleneck [9], evacuation from a building [34], etc. Furthermore, the agent-based model, a class of continuous models for simulating the actions and interactions of pedestrians, is upgraded in terms of flexibility, extensibility, and capability through a more general way [35,36].

This paper aims to investigate the stampede prevention design of Chinese primary school buildings, especially for the architectural layout from a macro perspective and the staircase design from a micro perspective. The next section makes an overview of materials and methods. Section 3 presents the relationship between the sustainable layout of crowded places (e.g., toilet and staircase) and the evacuation efficiency by agent-based simulations. Section 4 introduces the detailed analysis of the parameters of staircase design with experimental results to reduce the crowd stampede risk. Section 5 makes a detailed discussion and proposes suggestions for building layout and staircase design within the framework of sustainable building principles. Section 6 presents results for the stampede prevention design of Chinese primary school buildings and the future work.

## 2. Materials and Methods

According to official statistics, the per capita GDP and per capita disposable income in Guizhou were last recorded at 33,246 yuan and 1,512,115 yuan, respectively, in 2016. It is now a comparatively less developed area and eagerly waiting further construction in China. For the primary schools in this area, in addition to the energy conservation and cost savings, the sustainable safe environment should also be considered to stabilize the development process. Thus, three representative primary schools in Sandu (Guizhou) are selected as research objects and experiment sites to study the sustainable design of buildings. In particular, the overall parameters of ChengGuan Primary School are measured in detail for reference to build the spatial model. Based on the basic parameters such as the height, shoulder width and walking speed of teachers and students, this study employs the multi-agent software Pathfinder to simulate the daily scenarios in the primary school. Then, 72 students are randomly chosen as the experimental subjects to acquire the data of design parameters of the staircase.

### 2.1. Agent-Based Simulation

The sustainability of building design not only reflects the green environmental protection and energy conservation but should emphasize the fact that it can provide a safe and comfortable place for a long-term living [37]. Especially in an emergency, a building with the perspective of sustainable safety can ensure all pedestrians to be efficiently evacuated in time [38]. Namely, the proper layout and location arrangement of crowded places with constrained investment can contribute to the sustainable reduction in the crowd risk and reduce the total cost due to integrated design (e.g., locating the toilets in the building can get the lower cost of design and construction than putting them outside) [9]. With the special model, this paper discusses the impact of the key location layout (e.g., toilets and stairs) on the evacuation efficiency and safety state. Through the comparative analysis of risk indicators such as evacuation time, flow rate, and population density, optimized layout suggestions for primary schools considering safety and behavioral habits of students are proposed. 

#### 2.1.1. Parameter Design

Pedestrians can adjust their movements at any time according to the changes of environment. Pathfinder, a continuous agent-based software, can dynamically simulate the individual behavior and quantify the evacuation parameter such as the evacuation route and time with the graphics simulation technology. It can also provide abundant and intuitive process data such as heat map and 3D animation to present reliable references for actual evacuation drills and architectural design [39]. The software provides the continuous physical space during the evacuation, which can better fit the real-world human characteristics. Complete collision mechanisms are embedded in the system including the Society of Fire Protection Engineers (SFPE) and Steering mode. In general, the Steering mode is closer to the actual evacuation scenario and pedestrian behavior [40], which is adopted as the main method to simulate the evacuation scenarios. 

In the model, all pedestrians are assumed to be familiar with the exit of school, the position of staircases and the nearest exit is selected, regardless of the obstacles such as desks and lecterns in the classroom. Experiment results show that the walking velocity is not significantly affected by the gender and grade of pedestrians in the crowd evacuation (see Section 4.2). Thus, only two types of occupants are considered, regardless of the influence of gender and small changes in age. 45 students and eight teachers are randomly distributed in each classroom and office, with a total of 1144 people in the entire building. The parameter setting of adults refers to the in-depth research of Li et al. [10], and that of students refers to the findings of Najmanová et al. [41]. Meanwhile, the sample mean and SD of the experimental results are also employed, which considers the total time including the subtle pause and resultant velocity. The speeds of student and teacher are set to 0.8 m/s (±0.1) (Max: 1.55 m/s, Min: 0.56 m/s) and 1.19 m/s (±0.1) (Max: 1.56 m/s, Min: 0.85 m/s) respectively, which can be assigned automatically according to the different density based on the fundamental diagram of the software. Due to the individual difference, the shoulder width is set to 0.314 m (±0.02) and 0.368 m (±0.03) for students and teachers. The acceleration time and collision response time are both set to 1.3 s. 

#### 2.1.2. Spatial Model

**Figure 1:**
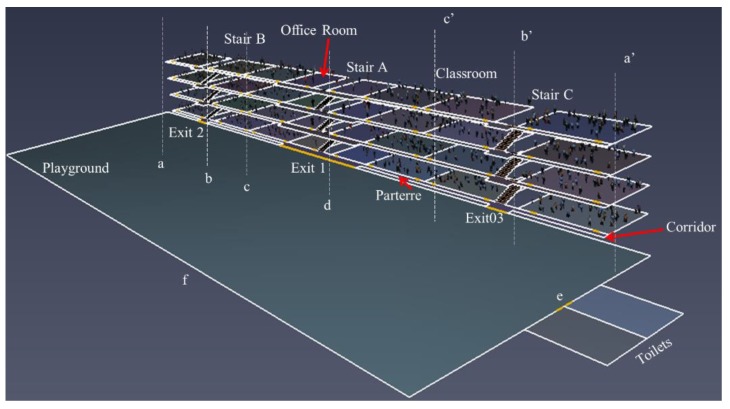
Special model for the ChengGuan Primary School.

Here we choose ChengGuan Primary School in Guizhou Province as an example. The overall layout of ChengGuan Primary School which represents the general design of most primary schools in China, is relatively neat. The spatial model is built according to its actual design size and layout (as shown in Figure 1). The four-story main building contains six classrooms (12 m × 8 m) and two office rooms (8 m × 8 m) on each floor. Each classroom can accommodate 45 students while the office rooms can contain eight teaching staff, the sex ratio of which is approximately 1:1. A corridor (97.6 m × 1.5 m) is next to the classrooms on each floor. Three staircases, indicated as Stair A, B, and C, (1.6 m × 0.3 m × 0.15 m) are distributed symmetrically on the middle, left, and right of the building. A single layer toilet (12 m × 8 m) is located on the side of the playground.

#### 2.1.3. Simulation Scenario

The crowd in elementary schools is relatively concentrated and follows the similar pattern of daily activity. For example, students go to school early in the morning and rush to toilets, small shops and the playground at the break. All the students do broadcast exercise during the morning break and then go home at noon and in the afternoon. The staircase plays an important role in multi-story buildings [42], meanwhile, it’s an area that is prone to crowd stampedes. Previous studies conducted special statistical analysis on primary schools and found that school hour, group activity and break periods are the times of high incidence of the CSA, and most of them are concentrated in the stairwell between the 1st and 2nd floor [1,42,43]. Fujiyama et al. [29] pointed out that the characteristics of the staircase are directly related to the safety of pedestrians, which vary according to the stair-gradient, visibility, the staircase dimension and the stair layout. The different distribution affects the direction and speed of the pedestrians’ movement.

Therefore, this study concerns the sustainable built environment. Two simulation scenarios (SS) are designed. SS 1 focuses on the entire evacuation activities like descending stairs after school, gathering activities, etc. Three sub-SSs are discussed as follows including the general layout of the primary school staircase. Pedestrians can evacuate through any exit of the building and the service capacity (total area) of staircases is the same in order to ensure that the total energy and economy consumption are equal in all sub-SSs [21].

- SS 1-1 (the existing layout): All exits and staircases are distributed in accordance with the actual situation and the location of side staircases is at “b-b’”;
- SS 1-2: Retain the position of Stair A and change the location of the side staircases (B and C) symmetrically to the positions “a-a’” (1-2-1) and “c-c’” (1-2-2), and the situation “d” (1-2-3) is simplified as a single staircase Stair A’ with a greater width;
- SS 1-3: Retain the position of Stair A and change the location of side staircases (B and C) asymmetrically to the position “a-b’” (1-3-1), “a-c’” (1-3-2) and “b-c’” (1-3-3).

SS 2 depicts the situation of dispersed crowd movements such as going to the toilet at the break. The toilet is a high incidence area for school crowd stampedes (e.g., the CSA mentioned in Section 1 occurred when students went to the toilets before sn examination). It has been discussed that the crowd flow within toilets varies due to their locations in reference to the entries of the main building [44]. The actual flow of pedestrians within each floor and the interflow between the adjacent floors may also affect the total service time. Thus, the distribution of toilets outside and inside the building are considered, particularly for the adjacent and separated toilet layout of different floors. The existing condition and other six sub-SSs representing the general design for the primary school are presented. It is assumed that about 20% of occupants can implement this action. As this study does not distinguish between genders, all pedestrians are free to use any toilet about 20 s, and then return to the original room. Three sub-SSs are included and pedestrians are free to choose any toilet. Note that the service capacity (the total area) of toilets is equal in all sub-SSs in order to make sure that the economic and environmental performance indicators of the sustainable built environment are the same [21]:

- SS 2-1: (the existing layout): two big toilets are distributed in the west of the playground;
- SS 2-2: two big toilets are located in the north of the playground and on the opposite side of the main building;
- SS 2-3: toilets are located every two floors alternately at the position “a”, two on the 1st and two on the 3rd (2-3-1) or two on the 2nd and two on the 4th (2-3-2);
- SS 2-4: four toilets are evenly distributed at the position “a” of each floor (2-4-1) or the different sides alternately (shown in Table 1).

### 2.2. Staircase Risk Experiment

Three staircases in the different primary school are selected as experimental sites and three experiment scenarios (ES) are designed for observation. According to the dynamic risk algorithm and the perspective of sustainable built safety, habitual behavior data extracted from the tracking process is adopted to calculate the design range of the staircase.

#### 2.2.1. Experimental Design

Seventy two healthy students from grade 1 to grade 6 (36 boys, 36 girls; age (mean ± SD):10 ± 1.5 y), all physically active, participated in this study. All subjects were screened to avoid significant skeletal or neurologic impairments and numbered from 1 to 72. The students are instructed on the procedures of the experiment and maintaining their daily walking posture to ensure the authenticity of the experimental results. The participants carried out the instructions to descend the staircase. The detailed structural data of the staircases at experimental sites including the riser height of each step (RH) *h*, step depth (SDP) *d*, step width (SW) *w*, the inclination angle (IA) θ are shown in Table 2. The participants are requested to walk at the speed of their habit while wearing light or flat shoes in order to achieve the data more consistent with the human ethology. Meanwhile, subjects are not allowed to use the handrail.

Three ESs are implemented at the experiment sites mentioned in Table 2. In the first ES, all participants are distributed randomly on the first step (the top floor). Then, participants receive a signal and move into the staircase one by one according to the experiment number. The movement of participants remains the normal condition under the consideration of safety. Then, they are requested to walk in pairs in the second ES and allowed to chat with each other. In the third ES, the evacuation and merging flow are considered to investigate the walking gait of students in emergencies. All participants begin to move into the staircase synchronously. To make participants more adaptable to different conditions, both repeated practice trials and video acquisition under the unconscious situation are obtained for later analysis. 

Each participant is involved in two trials and a five-minute break is allowed to maintain the authenticity of the experiments. The whole process is recorded by the preset electronic devices including four high definition cameras and two SLR cameras, which are placed at the rooftop, the bottom of the staircase and beside the 4th step [1]. 

**Figure 2:**
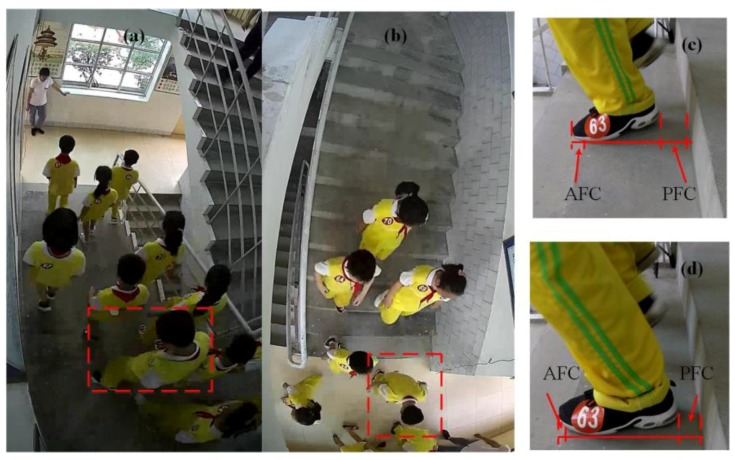
Snapshots of the evacuation process from the top step (**a**) to the ground (**b**) respectively, and the AFC and *PFC* on the 4th step in the ES 1 (**c**) and 2 (**d**).

The time period *T* that each student spends from the 1st to the 12th step can be extracted from the video frame which student holds the posture as shown in Figure 2 on the two steps. Then, the movement data such as the moving speed, the different conditions of posterior foot clearance (*PFC*) and anterior foot clearance (AFC) are obtained from the snapshots (shown in Figure 2). The actual values are calculated in proportion.

#### 2.2.2. Dynamic Risk Analysis

CSAs usually occur during the procedure of descending a staircase. The phenomenon of locomotion with one foot in contact creates a major challenge to our balance control system. Our total body mass is located on our single feet instantaneously if treating the entire process discretely. The inverted pendulum model [45] is adopted to calculate dynamic stability when descending the staircase. Previous studies have demonstrated the validity of the dynamic inverted pendulum model during human activities [46,47,48]. The margin of stability in the posterior direction is quantified by the difference between the threshold of the anterior (*pCOM*) and the extrapolated center of mass (*XCoM*). According to the geometry of staircase, the foot placement and step length are limited by the anterior edge of the step:

(1) $$\Delta COM=XCOM-pCOM=\frac{vCOM_{hor}}{\sqrt{gl^{-1}}}$$

where Δ*CoM* denotes the projection displacement of *pCoM*. *pCoM* indicates the anterior vertical projection of the CoM to the step. *vCoM_hor_* denotes the horizontal component of the participant’s speed. *g* is the gravity and *l* is the distance between CoM and center of ankle joint. 

**Figure 3:**
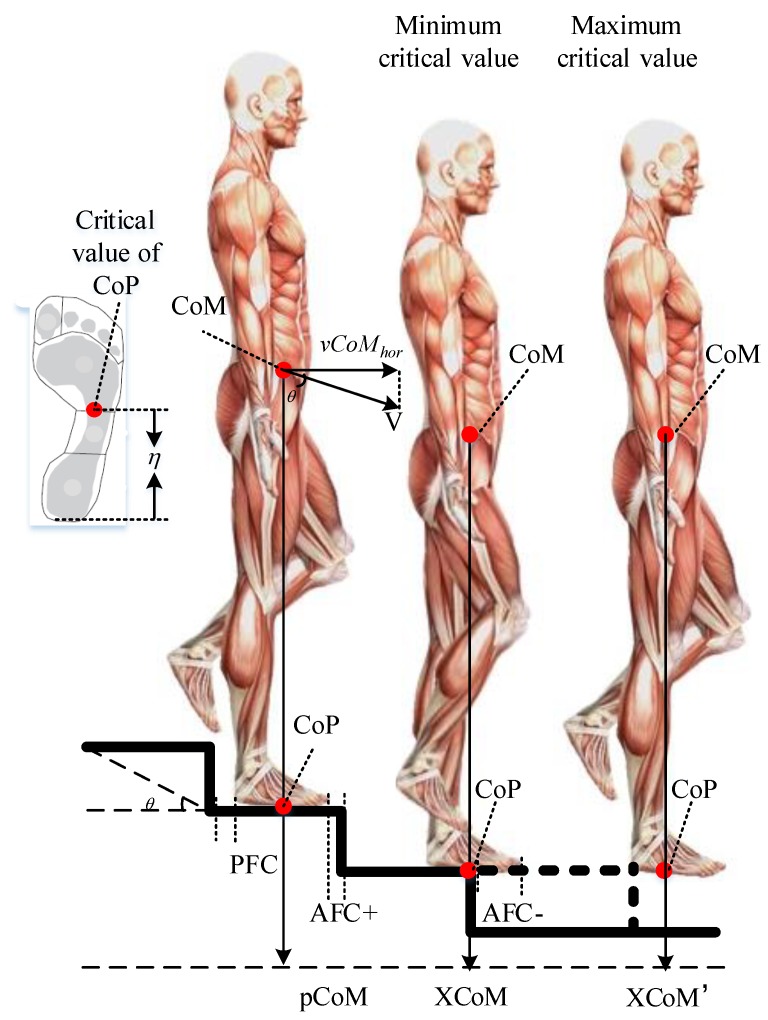
Inverted pendulum model while descending the staircase. Three discrete states to step down onto the next step are described, showing the vertical projection of the CoM, the horizontal initial velocity of the COM, the different conditions of *PFC* and AFC, and the tilt angle of the staircase. The middle posture repents the risk state and the minimum critical value of SDP. The right posture represents the maximum critical value of SDP.

In this study, the situation, as depicted in Figure 3 with the body slightly forward and one foot off the ground is treated as the discrete initial state to step down onto the next step. The CoM achieves an initial velocity *vCoM_hor_*. The margin of stability is usually determined in the anterior direction as the CSA generally occurs due to the sudden fall in front. Then, the postural risk is recognized if the position of *XCoM* exceeds the base of support (BoS) regarded as the edge of the anterior step. As the BoS is limited by the stair depth, participants tend to change the foot placement to adjust the center of pressure (CoP). Thus, at the critical state, the *XCoM* is located at the edge of step coinciding with CoP (see the middle instantaneous posture in Figure 3). 

Dynamic anatomic pressure data of foot is collected by Butterworth et al. [47] and the mask is developed to show six anatomical regions: whole foot, heel, midfoot, forefoot, hallux and toes (shown in Figure 3). The contact area and maximum force of midfoot and forefoot show the greater weight of multiple trials. Thus, the junction of these two areas is treated as the critical value of CoP and the distance between which and toes is expressed as *L* *− η*, where *L* is the foot length (FL). 

As mentioned above, we assume the movement of *pCoM* always satisfies the Equation (1). The left discrete posture in Figure 3 is regarded as the initial state on each step. *N* denotes the total number of steps. After descending *N* − 1 steps, the CoP of the participant should be still within the edge of the *N*-th step. Then the staircase is treated as safety design. The value of *d* is limited as follows:

(2) PFC+η+(N−1)×ΔCOM≤N×d

Furthermore, the excessively large value of *d* can also increase trouble and stress for people to decide whether he/she should dynamically adjust the gait or maximize his/her stride to drift with the current step. This creates a major challenge for control system while descending staircase. A “step-by-step” strategy according to the perceived distance is considered to be the most comfortable and effective gait. After descending *N* steps, the CoP of the participant cannot be within the range of the (*N* − 1)-th step. The upper limit value of *d* can be calculated as follows:

(3) (N−1)×d≤PFC+L+N×ΔCOM

Then, the design range of *d* can be presented as follows:

(4) PFC+η+(N−1)×vCOMhorgl−1N≤d≤PFC+L+N×vCOMhorgl−1N−1

## 3. Simulation Results: Stampede Prevention Design of Building Layout

The reasonable architectural layout is an important part of the architectural design with the sustainably built safety. The Pathfinder software is adopted to simulate the different SSs in this section. Two main layouts of staircases and toilets are discussed in different distributions and the indicators reflecting the evacuation efficiency are obtained from sub-SS simulations. The detailed analysis process and results are described as follows.

### 3.1. Stampede Prevention Layout of Staircases

Walking on the staircase is very common and important in the primary school life [14]. Considering the symmetry of the building, SS 1 includes seven common types of staircase distribution according to our investigations, which can basically represent the existing layout of staircases. The exits of the main building are limited by the parterres on the first floor. Under the premise that the exits in all sub-SSs meet the same total capacity, the total evacuation time, crowd flow, and the density of pedestrians near the key areas are compared, which can provide a sustainable layout reference for the staircase. 

**Figure 4:**
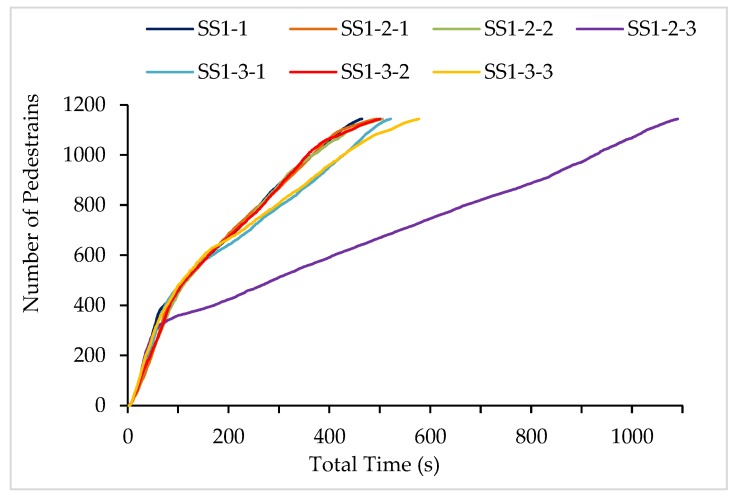
The number of occupants exiting from the main building under 7 sub-SSs.

In the seven sub-SSs, individuals need to evacuate through the three exits and reach the playground. Due to the different layout of staircases, the total evacuation time of pedestrians presents significant differences as shown in Figure 4, which is ranked as SS “1-1 bb’”, “1-2 aa’”, “1-3 ac’”, “1-2 cc’”, “1-3 ab’”, “1-3 bc’”, “1-2 d”. It can be recognized that the scattered layout of staircases achieves higher evacuation efficiency than that of centralized distribution, e.g., the total time of “SS1-2 d” representing single staircase condition is far greater than the other sub-SSs. The increase in the number of the staircase, rather than only widening the stair and realizing the centralized layout, can better enhance the evacuation efficiency. 

Meanwhile, the sub-SSs with the symmetric layout (e.g., “aa’”) which can also get a good appearance according to the aesthetic viewpoint show higher evacuation efficiency than that with asymmetric distribution (e.g., “ab’”). In the case of symmetrical distribution, the SS “bb’” shows a slight increase in evacuation efficiency than the SS “aa’”. Namely, the current layout presents an average population distribution for evacuation where the staircases divide the classrooms into four roughly equal parts. Then, the individuals reach the stairway on each floor with the shortest average distance. However, considering the limitation of actual condition or obstacles, the staircase cannot be always symmetrically distributed in some cases. According to Figure 4, the distribution of “bc’” and “ac’” show an earlier decline in slope and result of longer evacuation time. Relatively close positions of staircases, such as point b and c are already close to the middle stair, can easily cause mass gathering, thus forming high-density points and decreasing the evacuation efficiency.

**Figure 5:**
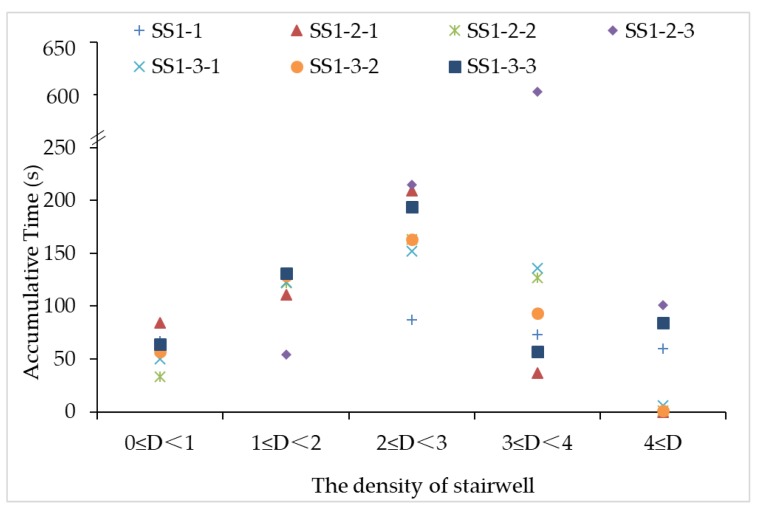
The accumulative time of different densities of the stairwell between the 1st and 2nd floor.

By reviewing the CSAs in the primary school, the stairwells between the 1st and 2nd floor are identified as the areas of high incidence of the crowd stampede risk. Thus, the maximum density and the accumulative time are calculated as the important indicators to show the evacuation efficiency in this area. Previous researches point out that it’s dangerous and the individual movement is extremely restricted when the crowd density exceeds 4 p/m^2^ (person per square meters). Figure 5 indicates that the “dd’”, “bc’”, and the current situation “bb’” hold higher density values than the threshold. The duration of high density in “dd’” contributes to a longer evacuation time and higher crowd risk. Similarly, with the continuous expansion of the distance between the two side staircases, the duration of high density decreases significantly. The separated staircase layout can make pedestrians move orderly with diverted crowd flow, avoiding the combination or superposition of high-density points. In the symmetrical distribution of SSs, the densities of stairwell are more evenly in all ranges and the average durations are lower. However, huge fluctuations of the accumulative time across different ranges occur in the SSs with asymmetrical distribution. The crowd asymmetrically flows into staircases and causes crowding in a certain door of the staircase. Overall, the situation “bb’” shows a relatively small density duration, which is approximately evenly distributed.

### 3.2. Stampede Prevention Layout of Toilets

Reasonable toilet distribution should be able to sustainably avoid the risk of CSAs while ensuring high service efficiency. The seven sub-SSs in the SS 2 representing the different layout of toilets in the primary school. The total execution time (TET), service time (ST), peak flow rate (PFR), and peak density (PD) can be calculated by the simulation results (shown in Table 3). The TET refers to the time that all pedestrians complete the task of going to the toilet and returning to the original classroom. While ST refers to the total time from the first pedestrian entering the toilet until the last one leaving. And both of the two indicators can reflect the service capability and efficiency of toilets. Additionally, to detect the evacuation safety of a building, special attention should be paid to the risk state (e.g., PFR and PD) at the most crowded situation, so that the safety of pedestrians can be effectively ensured in real emergencies. 

**Figure 6:**
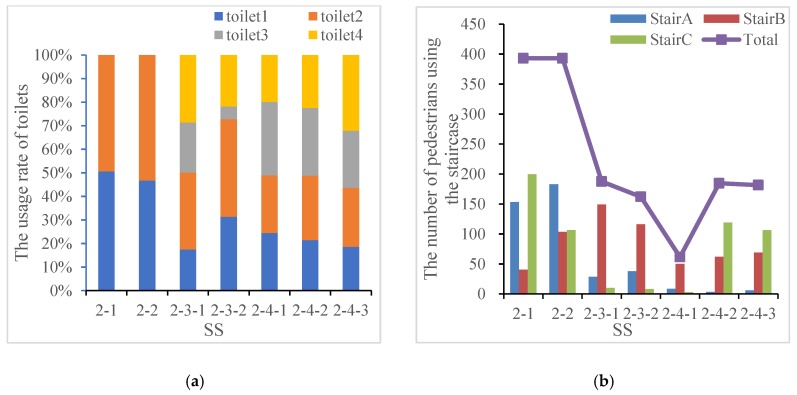
The usage of toilets and corresponding usage of staircases. (**a**) The usage rate of toilets in the SSs; (**b**) The number of pedestrians using the staircase.

Table 3 presents that the descending order of the service efficiency of toilets is SS “2-4”, “2-2”, “2-3”, and “2-1”, where the TET in SS “2-4-2” is 30% shorter than that in SS “2-1”. The difference in the relative position of the toilet near the playground and the main building has a greater impact on crowd movement. Meanwhile, the increase in the number of toilets significantly promotes the overall service efficiency. The shortest TET appears in the SS “2-4-2” and the other two sub-SSs in the “2-4” show relatively better evacuation efficiency than the other SS. In terms of the crowdedness, the PFR in SS “2-2” is the largest, whereas the maximum value of DP appears in SS “2-3-2”. Namely, placing toilets outside the main building increases the individual walking distance and reduces the waiting time, thereby reflecting a higher PFR at the toilet entrance; placing the toilets inside the main building decreases the average moving distance of pedestrians, but contributes to the crowd collision risk. In comparison, toilets in the SS “2-1” and “2-3” are less crowded. Therefore, from the perspective of stampede prevention design, the SS “2-4” and “2-2” seem to be able to better balance the service efficiency and crowd risk. In addition to the FRP and DP, the crowd flow of the staircase can also reveal some potential risks that are hidden in process of evacuation. A well-sustainable toilet layout should be able to evenly serve more pedestrians from all directions while minimizing the number of individuals using the staircases, thereby decreasing the risk of the CSA caused by bi-directional crowd flows. Figure 6a shows that toilets are used evenly in the SS “2-1”, “2-2”, and “2-4”. The two sub-SSs of the SS “2-3” indicate that pedestrians prefer to use low-floor toilets rather than climb to high floors. 

Comparing with the corresponding usage of staircases, Figure 6b shows that designing staircases inside the building greatly reduces the frequency of pedestrians using stairs. In regard to the SS “2-1” and “2-2”, pedestrians need to get down to the first floor and walk through the playground to the toilets, so the total number of pedestrians are equal on the staircases. As the toilets in the former situation are closer to Stair C and far away from the others, it reflects an uneven distribution of crowd flow. While the toilets are located at the central axis of the building in the latter scenario, the flow of pedestrians is symmetrical. 

When toilets are alternately distributed every two floors on the same side of the building (SS “2-3”), the use of toilets is not uniform. For the SS “2-3-1”, the usage rate of toilet 1 and 2 on the 1st floor is approximately equal to that of toilet 3 and 4 on the 3rd floor, but for the SS “2-3-2”, when the toilets are placed on the top floor, their usage drops sharply. The higher usage of toilets on the lower floor will inevitably lead to higher congestion. In addition, as Stair B is closer to the side toilets, the usage rate is consequently much larger than the other two staircases. However, in the real life, relevant ground facilities (e.g., drainage equipment, septic tank, etc.) are required, so it is generally guaranteed that there is a toilet on the first floor and the former design is better than the latter.

The SS “2-4-1” presents an extreme case that the side toilet on each floor satisfies the optimal choice of pedestrians separately. The crowd barely uses stairs and the trough appears in Figure 6b. The total usage of staircases in the SS “2-3”, “2-4-2” and “2-4-3” is roughly equal, but the latter two scenarios are more evenly, which can effectively balance the pressure of crowd flow. The usage of toilets on the middle two floors is greater than that on the bottom and top floors. This situation is more pronounced in SS2-4-2 because the toilet 2 and toilet 3 are adjacent and concentrated on one side of the building, which is more attractive to the pedestrians nearby. In all scenarios of “2-3” and “2-4”, the Stair A in the middle is used less frequently, which is more obviously in SS “2-4-2” and “2-4-3”.

## 4. Experiment Results: Stampede Prevention Design of Stairs 

The layout of staircases has an influence on the evacuation patterns of the crowd, but from a microscopic point of view, its design parameters also affect the safe walking of individuals [49]. From a perspective of the sustainable built environment, the mean value of two valid trials in the experiments is adopted for each participant. The differences are identified in the physical data of students from grade 1 to grade 6 such as age, height, the distance between CoM and center of the ankle joint (*l*) and FL (*L*). The movement data (e.g., PFD, AFD, total time, and walking speed) is extracted from the video footage with the guaranteed rate of 24 frames per second. The detailed analysis of student movement characteristics is shown in the following subsections.

### 4.1. Static Physical Characteristic

In the inverted pendulum model while descending stairs, *l* (*p* = 0.04) is treated as the length of pendulum directly affecting the amplitude of the swing. It is approximately measured as the distance between the navel and the ankle of students in the experiment reflecting a slow growth with height (*p* = 0.01) from grade 1 to grade 6 ranging from 0.754 m to 0.812 m (shown in Table 4). This tends to result in a higher anterior velocity and a more anterior displacement of *XCoM* for the advanced students. The relation between the height and *l* can be expressed by the regression $l=0.3484x_{heigh}+33.518$ ($R^{2}=0.73$) which will be discussed as the reference of sustainable staircase design in Section 5.2. Meanwhile, there are small differences in the critical values of COP within six grade students. According to Butterworth et al. [47], the junction of midfoot and forefoot is treated as the critical value of CoP, then, the value of *L*− *η* is calculated within the range of 0.21 m ± 0.09.

### 4.2. Movement Speed

Three ESs show respectively descending staircase alone, in a pair and together. In order to explore the characteristics in a different situation, the horizontal velocity of each student walking downstairs through all the steps is calculated using Equation (5) [42]. At the same time, individuals may have a pause in a certain step to compromise the anterior behaviors, especially in the crowd evacuation:

(5) $$vCom_{hor}=\frac{Nd}{T-Tp}$$

where *Tp* is the total value of subtle pauses on the certain step. The movements of COM while the student descending stair alone and in a pair are treated as continuous actions (*Tp =* 0 s). However, the student’s movement in ES 3 is regarded as a discrete process and the pauses are removed from the total time due to the influence of the surrounding crowd.

**Figure 7:**
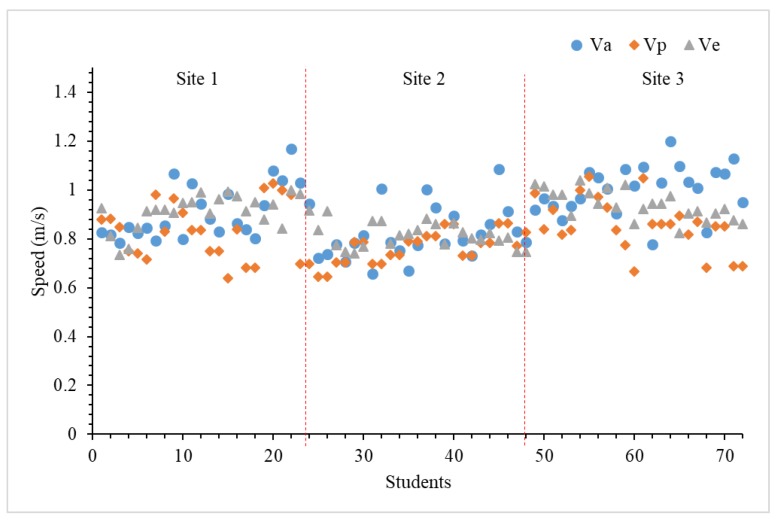
The speed of each student walking through 12 steps for ESs.

The velocities of students descending staircase alone (Va), in a pair (Vp), and evacuation (Ve) are shown in Figure 7. It indicates that there are great differences among students traversing the staircase alone due to individual characteristics. Students are more active and uncontrollable compared with adults [42]. Abnormal behaviors (e.g., walking across steps, playing games, picking things, inclining the body, etc.) frequently appear in the independent walk, which affects the overall speed. The Vps of students in ES 2 present a similar trend with Va, and obviously, equal velocity pairs appear with larger discrete characteristics. The students in ES 3 travel at approximately the same speeds except for some leaders which enter the stairs first. Namely, when an individual is involved in a group, he or she cannot select his or her prefer moving velocity, but can only drift with the crowd. The individual’s movement is treated as discrete in this ES. The multiple pauses of individuals in order to compromise the anterior students are removed from the total evacuation time.

**Figure 8:**
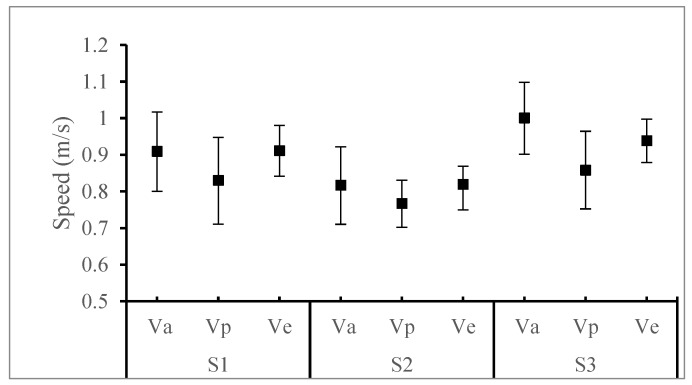
The mean speeds for all students walking through 12 steps for three ESs at three sites.

Figure 8 presents the mean speeds and SDs for all students within the ESs at the three sites. The mean speed of the individual shows a dramatic decrease with increasing number of peers. The Va at site 3 is the highest with the value of 1.0 m/s (±0.1) and the other two sites reflect relatively lower speeds with 0.91 m/s (±0.11) and 0.82 m/s (±0.1). It indicates that speeds differ in individual characteristics, surrounding environments, age, and gender, etc. [1], but roughly cover the range 0.91 m/s (±0.13), according to the sample mean and SD. Similarly, the speed of descending in pairs presents personal differences at the three sites. Participant’s attention is distracted by the communication, contact action, mutual gaze, interplay, etc. Then, the descending movement is obviously delayed, so that the Vps at site 1 (0.83 m/s ± 0.11), site 2 (0.76 m/s ± 0.06), and site 3 (0.86 m/s ± 0.11) are significantly lower than the Vas, separately. However, the mean descending speed of students in the ES 3 at different sites where the design parameters of the staircase are floating in a small range is almost equal. Therefore, we can assume that when the other parameters are controlled, a small change in the SDP will not have a significant impact on the overall speed.

The CSA often occurs in the condition of mass gathering like the ES 3. The above two ESs are relatively safe because individual walking has greater security and buffer space, and the crowd density around them is relatively small. However, it’s difficult for students to overtake others while descending stairs. The faster individuals in ES 1 need to slow down their speed and pause to compromise with the anterior students. The slower students form bottlenecks to limit the continuity and efficiency of evacuation, which may easily trigger the CSA under certain condition. Due to the evacuation scenario and space constraint, Ve exists in the range of Va and Vp. Thus, the sample mean value of Ve (0.88 m/s ± 0.08) is selected as the target $vCom_{hor}$ which is more consistent with the subject of stampede prevention design.

### 4.3. Gait Characteristics

The gait characteristics of students while descending the staircase have a significant influence on the stampede prevention design of SDP. The values of *PFC* and AFC are proportionately extracted from the video frames of specific discrete states and the mean values from valid trials are calculated from the two ESs (descending alone, PFCa and AFCa, descending in a pair, PFCp and AFCp). In particular, due to the overcrowding in ES 3, the gait characteristics of different individuals within the video frames are overlapped and difficult to observe. Thus, the first two ESs are considered to obtain the gait characteristics of students.

**Figure 9:**
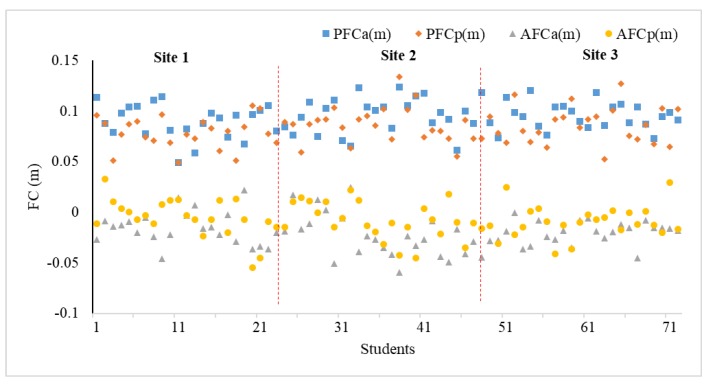
The FC of each student walking through 12 steps for two ESs.

When going downstairs, due to obtaining a forward horizontal initial velocity, the trajectory of the anterior foot is often not a vertical motion, but a horizontal projectile motion (a < g). There may be a distance between the vertical “riser” and the anterior foot placement for individuals to adjust their body balance. Results in ES 1 and 2 as shown in Figure 9 verify the hypothesis that the gap (*PFC*) does exist, which is roughly estimated to be centrally distributed around 0.1 m. Meanwhile, the PFCs of students in two ESs show a similar distribution which does not appear large flotation according to the construction of the staircase. Then, it is inevitable that individual’s toes exceed the outer edge of the step and the negative value of AFC will occur. The majority of AFC of students are below 0 discretely distributing with respect to the SD of FL.

**Figure 10:**
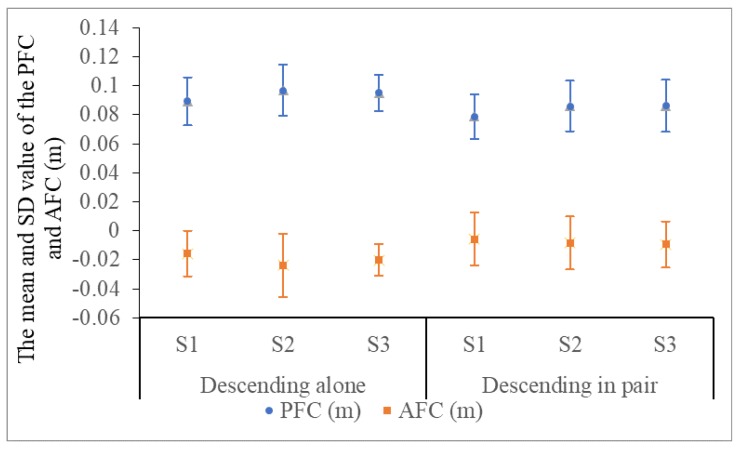
The mean value and SD of FC for all students in two ESs at three sites.

Furthermore, Figure 10 introduces the mean value and SD of FC for all students descending stairs alone and in a pair. The mean value of PFCa at sites 1 (0.089 m ± 0.02), sites 2 (0.097 m ± 0.02), and sites 3 (0.095 m ± 0.02) decreases separately with adding a companion contrasting with PFCp at three sites (0.079 m ± 0.02, 0.086 m ± 0.02, and 0.086 m ± 0.02). The reason for narrowing the distance might be that the students divert their attention to communicate with peers and voluntarily draw back his or her *XCoM* to ensure a larger contact area of the anterior foot to reduce risks. Namely, with the increase in the number of peers, people spontaneously reduce the value of *PFC* according to their habits, especially in the crowd evacuation. Thus, in order to be more consistent with the sustainable individual preference and close to the situation of descending the staircase together, the sample mean value of PFCp (0.084 m ± 0.02) is selected as the reference of *PFC*. Relatively, the pedestrian habitually moves the front foot beyond the edge of the steps (−0.02 m ± 0.02) and withdraws in the case of descending in a pair (−0.008 m ± 0.02). Considering the environmental loss and minimizing the risk of the CSA, the initial value AFC is set to zero. We only consider the influence of *PFC* on SDP and take the AFC as an indicator of the initial state.

### 4.4. The Design Range of SDP

Assume that all parameters generally satisfy the normal distribution, while subjects (*N* = 72) serve as a sample of the normal population. According to the interval estimation of the normal population, the overall estimation of parameters is calculated by $v\bar{x}\pm t_{\frac{\propto }{2}}\left(n-1\right)\frac{s}{\sqrt{n}}$, where the confidence level α is set to 0.05. The floating range of the parameters mentioned in Equation (4) and the design range of SDP are shown in Table 5. 

$vCom_{hor}$ has the great impact on SDP, and an increase of 0.03-unit speed brings a 0.01 m design deviation, which means that faster walking speed requires a wider SDP to reduce the risk of the CSA. *l* has a significant impact on the design of SDP, creating a gap of 0.006 m to the maximum and minimum values. This also shows that since students are much lower than adults in height, the design of the staircase in primary school should be considered separately rather than the conventional design. *PFC* and *η* show less influence on SDP compared with the other parameters. Considering saving the construction materials, *N* is set from 7 to 12 as the number of consecutive steps is generally designed from 10 to 12 based on the investigation. The minimum value of *N* is reduced in order to obtain a more general design range. Then, the minimum critical value of SDP is calculated as the maximum value of the left ten subranges and the maximum critical value is regarded as the minimum value of the right ten subranges using Equation (4). Then, the stampede prevention design range of primary school buildings is obtained from 0.250 m to 0.282 m.

## 5. Discussion

The simulation and experimental results show that different design schemes bring different levels of risk to buildings. Furthermore, how to combine the policy background of sustainable development in China with the concept of a sustainable built environment is to be discussed henceforth. The range of SDP in the primary school is accurately calculated based on the perspective of stampede prevention and sustainable use.

### 5.1. Sustainable Building Layout

The government has already issued the general building code of the primary school. However, due to factors in the different region such as the level of economic development, regional culture, and special landform, various types of building layout appear in the primary school of different areas in China. The concept of sustainable building layout to these areas may lead to the different emphasis, as it not only needs to meet the regulation of environment and regional culture but the economic restriction.

Three primary schools in Guizhou, a less developed province in China, are selected as our research subject schools, one of which is in the town (the simulation sample) and the other two are in the countryside. The latter’s funds for construction are often short, which are often one-time government investments or corporate sponsorships, so their building concept tends more to consider economic sustainability firstly, such as building a small number of staircases and one toilet on the first floor. However, this situation greatly increases the risk of the stampede and most CSAs occur in primary and secondary schools in underdeveloped areas. Thus, in the case of limited construction materials and funds, the toilets should be placed near the central axis of the main building, then the total distance from each exit can be minimized. Meanwhile, staircases should be placed on the division points of the classrooms on each floor. 

With regard to the primary schools in the developing regions such as our target school, Chengguan Primary School, occupy more teaching and government resources. It should involve a sustainably built environment perspective and take the stampede prevention design into account. The staircases in the current situation show higher evacuation efficiency and average population distributions. However, if the idea of saving energy and cost is adopted to concentrate all staircases into one, the evacuation efficiency should be greatly reduced. The current placement of toilets (constructing outside the teaching building) is a way of wasting resources and increasing the overall mobility of the students. To avoid long duration of congestion, it is more sensible to set up the toilets on the same side and distribute them on each floor, which is more conducive to the unified design and construction of the drainage, piping and green facilities in the early stage. This can effectively share the pressure and reduce the risk of the CSA due to the crowd moving across floors. It is worth mentioning that the design of double toilets on both sides per floor is, of course, the best choice if the funds are sufficient. If designed every two floors alternately, toilets should be located on the first floor in terms of practicality.

### 5.2. Sustainable Design of the Staircase

**Figure 11:**
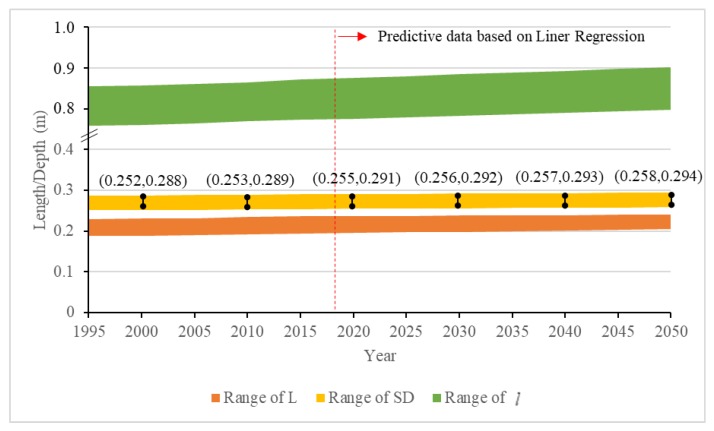
The historical and predictive range of L, SDP and *l*.

Apart from the overall layout of the building, the staircase, as an area of high incidence of the CSA, is discussed in detail. Section 4.4 presents the accurate design range of SDP based on the reference values obtained through experiments and observations. Equation (4) presents the method of calculating the limit value to prevent people from falling down while going downstairs at the present time. However, the physical data such as height and FL of primary school students develop slightly according to the historical and predictive data (shown in Figure 11). The dynamic changes of these indicators have a profound influence on the actual walking gait and the design of SDP in the future. Thus, with a sustainably built environment perspective, the growth of physical characteristic should be also taken into account for the sustainable prevention of the CSA.

Based on the height and FL records of students between 7 and 12 years old in China, the linear regression equations are set up to predict the growth intervals in future ($y_{heigh}=0.4359x+78.033,\;R^{2}=0.98$; $y_{FL}=0.1629x+18.208,R^{2}=0.97$ ). The values of *l* are obtained using the regression equation mentioned in Section 4.1. Figure 11 clearly presents that the dynamic changes of SDP increase at a rate of 0.001 m per ten years. The difference between the maximum and the minimum is basically stable at 0.36 m. It is difficult to make late adjustments for the building structure once it is built as a huge asset. Therefore, certain reserve values should be added to the design parameters of SDP for long-term use. We assume that the service life of the building is more than 30 years, then the forecasting range of SDP is about 0.258 m to 0.294 m. Considering the current building restrictions, the stampede prevention design of SDP in Chinese primary school is finally determined to be in the range of 0.258 m to 0.282 m.

Meanwhile, the possible larger value in this interval should be given priority in reducing the possible risks. According to the dynamic risk analysis, the CoM should be within the BoS to ensure that the total body mass may not exceed the CoP of the single feet instantaneously if treating the entire process discretely. However, when the limbs perform instructions from the brain, there will be subtle behavioral deviations due to emotional and environmental factors, which is also an important reason that distinguishes people from machines. This can be also proved by the differences in the observational data from multiple trials with the same participant. Thus, the larger value of SDP in the interval (0.258 m, 0.282 m) is selected to offset the possible risks of behavioral deviations.

## 6. Conclusions

This study investigates the sustainable built environment according to the habitual daily scenarios of students to propose the stampede prevention design of Chinese primary school buildings. Then, the precise range of SDP is calculated considering the dynamic steady ability and the growth of physical characteristics.

In the simulation, all pedestrians evacuate from the seven sub-SSs of different staircase distribution. The evacuation parameters are analyzed from the number of occupants exiting from the main building and the accumulative time of different densities. It is found that the symmetrical distribution of staircase design that occupies the division points of the classrooms on each floor can effectively share the evacuation pressure, and thus avoid the risk of the CSA. Meanwhile, the other seven sub-SSs of toilet layout is carried out. The evacuation efficiency and risk state of toilets are analyzed. It’s observed that the toilets should be placed on the same side of each floor, which has important practical significance for improving the efficiency of toilet usage and reducing the risk of the CSA.

In addition, 72 healthy students were required to carry out the instructions to descend the staircase in three ESs at the three sites. The static physical characteristics, movement data, and gait characteristics are extracted from the measurement and video frames. Based on the proposed dynamic model and historical data, the interval (0.258 m, 0.282 m) is finally determined as the stampede prevention design range of SDP. The larger value in the interval is suggested to be selected in order to decrease the risk of behavior deviations.

However, it should be noted that the participants in our design are primary students in less developed regions. The investigation and experiment data cannot represent the general features of all primary schools in China but shows that representative schools urgently need to be redesigned or developed. Moreover, the students in our experiments are much more active than adults and the evacuation scenario is hypothetical rather than the real emergency. Therefore, more experiments of the movement on the staircase for students should be conducted trying to approach the real emergency situations.

## Figures and Tables

**Table 1:** The staircase and toilet distribution in different SSs. “●” means the staircase is located at the position and the toilets in the SS 2 are numbered by “①”, “②”, “③”, and “④” (shown in Figure 1).

SS1	Staircase Distribution							SS2	Toilet Distribution									
	a	b	c	a’	b’	c’	d		1st		2nd		3rd		4th		e	f
									a	a’	a	a’	a	a’	a	a’		
1-1		●			●		●	2-1									①②	
1-2-1	●			●			●	2-2										①②
1-2-2			●			●	●	2-3-1	①②				③④					
1-2-3							●	2-3-2			①②				③④			
1-3-1	●				●		●	2-4-1	①		②		③		④			
1-3-2	●					●	●	2-4-2	①			②		③	④			
1-3-3		●				●	●	2-4-3	①			②	③			④		

**Table 2:** Detailed data of experiment sites.

Experiment Site	The Experiment Number of Students	Staircase Design				
		RH (m)	SDP (m)	SW (m)	IA (°)	Number of Steps
Gugua Primary School	1 to 24	0.15	0.30	1.6	26.57	12
Zhongle Primary School	25 to 48	0.15	0.29	1.45	27.35	12
Chengguan Primary School	49 to 72	0.15	0.28	1.36	28.18	12

**Table 3:** The evacuation efficiency and risk state of toilets in the SS 2.

SS	sub-SS	TET (s)	ST (s)	PFR (*p*/*s*)	PD (*p*/*m*^2^)
2-1		530	297	1.42	0.73
2-2		445	266	1.47	1
2-3	2-3-1	501	296	1.4	1.1
	2-3-2	587	384	1.36	1.79
2-4	2-4-1	408	234	1.33	0.79
	2-4-2	360	237	1.36	1.375
	2-4-3	382	254	1.26	1.21

**Table 4:** Static physical characteristics of the single support phase for each grade student (*n* = 12) while standing beside stairs (mean ± SD).

Physical Characteristic	Grade 1	Grade 2	Grade 3	Grade 4	Grade 5	Grade 6	Total	*p*
Age	8.0 ± 0.0	9.0 ± 0.0	10.1 ± 0.3	11.1 ± 0.7	11.3 ± 0.8	12.1 ± 0.3	10.3 ± 1.5	0.00
Shoulder Width (m)	0.286 ± 0.02	0.296 ± 0.01	0.305 ± 0.01	0.315 ± 0.01	0.324 ± 0.02	0.356 ± 0.02	0.314 ± 0.02	0.00
Height (m)	0.121 ± 5.3	0.127 ± 0.03	0.132 ± 0.04	0.140 ± 0.05	0.148 ± 0.07	0.151 ± 0.08	0.136 ± 0.01	0.01
*l* (m)	0.75 ± 0.03	0.778 ± 0.03	0.795 ± 0.05	0.815 ± 0.03	0.855 ± 0.02	0.865 ± 0.02	0.812 ± 0.09	0.04
*L* (m)	0.216 ± 0.02	0.215 ± 0.02	0.225 ± 0.02	0.224 ± 0.01	0.229 ± 0.17	0.238 ± 0.01	0.224 ± 0.02	0.02

**Table 5:** The floating range of parameters and the design range of SDP.

Parameter	Sample Mean (±SD)/Range	Interval Estimation/Range	SDP (m)			
			Minimum		Maximum	
			Min	Max	Min	Max
*PFC* (m)	0.08 ± 0.02	(0.075, 0.085)	0.242	0.245	0.296	0.330
*η* (m)	0.11 ± 0.01	(0.108, 0.112)	0.245	0.246	0.295	0.297
$vCom_{hor}$ (m/s)	0.88 ± 0.08	(0.711, 0.745)	0.241	0.250	0.291	0.302
*l* (m)	0.81 ± 0.09	(0.789, 0.831)	0.242	0.248	0.293	0.300
*N*	7 to 12	(7, 12)	0.242	0.245	0.296	0.329
Total (Max(Min), Min(Max))			**0.250**		**0.282**	
